# Effect of Gelatin Concentration on Humidity-Dependent Film Properties and Coating Performance: A Case Study of Vacuum-Packaged Sliced Cooked Cured Pork Loin

**DOI:** 10.3390/polym18212790

**Published:** 2026-11-08

**Authors:** Dariusz Kowalczyk, Karolina Wójciak, Paulina Kęska, Monika Basiura-Cembala, Tomasz Skrzypek

**Affiliations:** 1 Department of Biochemistry and Food Chemistry, Faculty of Food Science and Biotechnology, University of Life Sciences in Lublin, Skromna 8, 20-704 Lublin, Poland; 2 Department of Animal Food Technology, Faculty of Food Science and Biotechnology, University of Life Sciences in Lublin, Skromna 8, 20-704 Lublin, Poland; karolina.wojciak@up.edu.pl (K.W.); paulina.keska@up.edu.pl (P.K.); 3 Institute of Engineering Sciences, Faculty of Materials, Civil and Environmental Engineering, University of Bielsko-Biala, Willowa 2, 43-309 Bielsko-Biala, Poland; mbasiura@ubb.edu.pl; 4 Department of Biomedicine and Environmental Research, Faculty of Natural Sciences and Health, The John Paul II Catholic University of Lublin, Konstantynów 1J, 20-708 Lublin, Poland; tomasz.skrzypek@kul.pl

**Keywords:** gelatin film, edible coating, relative humidity, oxygen permeability, meat products, lipid oxidation, total viable count

## Abstract

This study evaluated gelatin (GEL)-based edible coatings as a complementary preservation approach for sliced cooked cured pork loin (CCPL) vacuum-packaged in polyamide/polyethylene (PA/PE) film and stored at 4 °C for 21 days, focusing on the effect of GEL concentration (5% vs. 10%) on film properties and coating performance. GEL films were characterized at 40–90% relative humidity (RH), with PA/PE film as a benchmark. Despite the more compact cryo-SEM microstructure of the 10%-GEL film/coating-forming solution network, the resulting films showed no significant differences in microstructure, crystallinity, or most physicochemical properties. Compared with the PA/PE, GEL films exhibited a more compact structure, higher transparency, stronger UVB/UVC-blocking capacity, lower oxygen permeability at 40–60% RH (127.75–1231.25 vs. 4234.06–5056.72 cm^3^ μm m^−2^ d^−1^), and higher puncture strength at RH ≤ 70% (9.12–15.24 vs. ~6.6 MPa). Increasing RH elevated oxygen permeability in all films and moisture uptake in GEL films, thereby reducing their mechanical strength. Coating application was associated with reduced oxidative stability and increased microbial counts, with effects varying by GEL concentration and storage time. These findings demonstrate that favorable film properties measured under standard conditions do not necessarily translate into effective preservation of meat products when employed as edible coatings.

## 1. Introduction

The resource-intensive nature of meat production, coupled with substantial meat waste [1,2], underscores the need for strategies to extend meat shelf life and enhance the sustainability of the meat industry. This is particularly relevant for sliced meat products, where slicing and handling increase exposure to atmospheric oxygen and may promote lipid oxidation, which can continue after vacuum packaging due to residual oxygen and oxygen permeation through the packaging material. Equally importantly, the increased surface area of sliced meat products may promote microbial spoilage. In response to these challenges, various protective hydrocolloid-based edible coatings have emerged as a promising approach that can be integrated with conventional packaging systems [3,4,5,6,7,8,9].

Understanding the behavior and potential functionality of coating materials in food applications requires characterization of their physicochemical properties, including water affinity, barrier properties, optical characteristics, and mechanical performance. Free-standing films provide a standardized platform for assessing these intrinsic properties and understanding structure–function relationships under controlled conditions. However, film properties may not fully reflect coating behavior in situ, where interactions with the food matrix and storage conditions can modify their structure and functionality. Thus, characterization of free-standing films provides complementary information for interpreting coating performance, which ultimately requires direct evaluation on the target food matrix [10].

Glazing meat with gelatin (GEL) is a traditional technique used in the preparation of aspics, galantines, and other jellied meat products, providing a glossy appearance, reducing moisture loss, and forming firm, decorative structures. Since the 1950s, edible GEL coatings have been extensively studied to extend the shelf life of various meat systems [11]. Their effectiveness has been shown to depend heavily on the food matrix and coating formulation. On vacuum-packaged beef, a 10% GEL coating provided less antimicrobial and antioxidant protection than chitosan [12], while GEL/chitosan blends showed concentration-dependent effects on lipid oxidation and color [13]. In fresh meat, a 20% GEL coating reduced purge loss but did not significantly affect lipid oxidation or flavour and negatively affected color [14]. A 5% GEL-based coating containing carboxymethyl cellulose improved selected quality attributes of vacuum-packaged sliced sausages [15]. The effect of GEL concentration has also varied among products: 7.5% GEL provided better overall preservation of chicken patties than 5% or 10% GEL, whereas 10% GEL provided better control of weight loss and bacterial growth than 5% GEL in beef meatballs [16,17]. Similarly, 4% and 6% GEL provided greater protection against oxidation and color changes in cooked ham and bacon than 2% GEL, although differences between 4% and 6% were not always significant [18]. These findings indicate that the effects of GEL concentration are product-dependent and cannot be reliably extrapolated across different meat products.

Although GEL concentrations used for film and coating formation range from 1% to 20% [19], concentrations below 5% may prolong drying, whereas those above 10% may cause excessive viscosity and premature gelation, hindering uniform coating [16]. Therefore, in the present study, 5% and 10% (*w*/*w*) GEL were selected as two contrasting concentrations within a practically feasible processing range, allowing the effects of a twofold increase in GEL content to be evaluated.

Biopolymeric coatings can serve as complementary protective layers within conventional packaging systems (particularly after package opening) rather than as replacements for conventional packaging. If they provide sufficient additional protection, they could potentially contribute to reducing plastic use by enabling thinner packaging films or reducing reliance on complex composite materials [9]. This is particularly relevant to polyamide/polyethylene (PA/PE) films, which are widely used for vacuum- and modified-atmosphere packaging of meat but are difficult to recycle because of their multilayer structure [20]. In PA/PE films, the polymer layers provide complementary functions: PA primarily provides oxygen barrier and mechanical strength, whereas PE provides moisture resistance and heat-sealing functionality [21]. In this context, comparing GEL-based films with PA/PE film can identify differences in their functional properties and clarify the potential complementary role of GEL within existing packaging systems. Previous studies have shown that, compared with other biopolymers, GEL plasticized with glycerol or sorbitol forms mechanically strong, stiff, highly transparent, and UV-blocking films [22,23,24,25]. However, these properties were typically determined at 25 °C and 50% relative humidity (RH), conditions that do not represent the high-humidity environment of meat packaging. Like other hydrocolloid-based films, GEL films are highly moisture-sensitive, meaning that water uptake can substantially alter their properties [26]. Notably, RH can also affect the barrier properties of conventional packaging materials [27]. Therefore, meaningful comparison of GEL and PA/PE films requires characterization across a range of RH conditions relevant to their intended application.

Although GEL concentration strongly affects solution viscosity, gelation kinetics, and network formation [28], its direct effect on film and coating performance remains insufficiently understood. In particular, previous studies [29] reported increased mechanical strength and reduced water vapor and oil permeability with increasing GEL concentration (4–8%), but differences in film thickness prevented these effects from being attributed solely to concentration. Moreover, the effect of GEL concentration on oxygen permeability (OP), a critical parameter for the protective performance of food packaging films, remains unresolved [29]. Therefore, standardized film thickness is essential for isolating the effect of GEL concentration on film properties. Moreover, it remains unclear whether concentration-dependent differences in the humidity-dependent properties of free-standing GEL films, particularly oxygen permeability, translate into differences in coating performance on high-moisture portioned meat products that are typically vacuum-packaged.

Against this background, this study aimed to evaluate the effects of GEL concentration on film properties and coating performance, using vacuum-packaged sliced cooked cured pork loin (CCPL) as a case study. Specifically, the objectives were to: (i) compare the properties of film/coating-forming solutions (FCFSs) containing 5% and 10% (*w*/*w*) GEL; (ii) characterize the structural, physicochemical, barrier, mechanical, and antioxidant properties of the corresponding films conditioned at 40–90% RH, using PA/PE film as a practical benchmark; and (iii) evaluate quality changes in CCPL slices coated with 5% and 10% GEL FCFSs, vacuum-packaged in PA/PE bags, and stored at 4 °C for 21 days. By comparing humidity-dependent film properties with quality outcomes in CCPL, this study explores their relevance to coating performance in a specific food–packaging system.

## 2. Materials and Methods

### 2.1. Materials

The FCFSs were made from a porcine GEL (type A, Bloom strength of 240, Kamis; McCormick Polska S.A., Stefanowo, Poland) and glycerol (Sigma-Aldrich, St. Louis, MO, USA). *Musculus longissimus lumborum* (pork loin) was collected from commercial crossbred pigs slaughtered at approximately 6 months of age, with a live body weight of 110–120 kg. The muscle was excised 24 h postmortem. Commercial PA/PE bags (20 × 40 cm) were used for packaging.

### 2.2. Preparation and Analysis of FCFSs

Aqueous solutions containing 5% or 10% (*w*/*w*) GEL and glycerol (20%, *w*/*w*, based on the GEL mass) were heated at 90 °C for 30 min and then cooled. Cryogenic scanning electron microscopy (cryo-SEM) observations were performed using an Ultra Plus scanning electron microscope (Carl Zeiss, Oberkochen, Germany). The pH of the FCFSs was measured at ~25 and ~40 °C using a glass ERH-11S electrode connected to a CPC-401 pH meter (Elmetron, Zabrze, Poland). The color parameters (L*, C, and h) were measured using an NH310 colorimeter equipped with a 5 × 5 × 5 cm liquid sample accessory (3nh, Guangzhou, China). The opacity (Opa, A_600_/mm) was determined using a Lambda 40 spectrophotometer (PerkinElmer, Shelton, CT, USA) as the absorbance at 600 nm divided by the optical path length (10 mm), with distilled water used as the blank. Dynamic viscosity (η, mPa·s) was determined using a ROTAVISC lo-vi rotational viscometer (IKA, Staufen, Germany) under the following operating parameters: SP-1 spindle, 200 rpm, 40 °C, 400 mL of the sample, with the reading taken after 3 min. The gelation time (t_gel_) of the FCFSs was determined using ball-drop method [30], with slight modifications. Briefly, the FCFS (30 g, 35 °C) in a glass beaker (50 mL) was left at room temperature (~25 °C) to cool down. A metal ball (3 mm in diameter, ~0.11 g) was dropped into the FCFS at one-minute intervals as the solution began to lose fluidity until the ball became immobilized in the gel. Additionally, a gelation temperature (T_gel_) was recorded. The beakers containing the resulting gels were tightly covered and kept at 25 °C for 24 h. Then the gel strength (GS), corresponding to maximum force (g) required for the 5 mm diameter ball probe (P/5S) to penetrate 10 mm into the gel at a speed of 1 mm/s, was measured with a texture analyser TA.XTplus (Stable Micro Systems, Godalming, UK). The analyses of the FCFSs were performed at least in triplicate.

### 2.3. Preparation and Characterization of GEL Films

The degassed FCFSs were cast on polycarbonate trays (144 cm^2^) and dried on a leveled heating table (~30 °C, 50% RH, ~16 h). A constant mass of non-water components (0.0125 g/cm^2^) was placed onto the trays in order to maintain ≈90 μm film thickness (namely, 30 g of the 5%-GEL and 15 g of the 10%-GEL FCFSs were cast onto trays). The films were peeled off and cut into samples of various shapes, depending on the test.

Thickness of the samples was determined using a Mitutoyo 547-401 digital thickness gauge (Mitutoyo, Tokyo, Japan). Before testing, the film samples were conditioned in an MLR-350 climatic test chamber (Sanyo Electric Biomedical Co., Ltd., Osaka, Japan) for 48 h at 25 °C and 40–90% RH.

The air-side surface and cryofractured cross-section of the films (prepared after immersion in liquid nitrogen) were analyzed using an Ultra Plus scanning electron microscope (Carl Zeiss, Oberkochen, Germany). Prior to SEM observation, the film samples were sputter-coated with gold. SEM images were acquired at 3–5 kV with a working distance of approximately 12 mm using a secondary electron detector (Carl Zeiss, Oberkochen, Germany). Additionally, the morphology was visualized using a Leica DM5500 B microscope (Leica Microsystems GmbH, Wetzlar, Germany) equipped with a differential interference contrast (DIC) optical system. Wide-angle X-ray diffraction (WAXD) patterns of the films and reference materials, including commercial low-density polyethylene (LDPE) and two polymorphic forms of PA6 (α and β), were obtained on a URD 6 Seifert X-ray diffractometer (FPM-Seifert, Freiberg, Germany) with a Cu Kα radiation source under the following operating conditions: 30 mA, 40 kV, scanning speed of 0.1° per 15 s, step size 0.1°, and ~25 °C. Color parameters (L*, a*, and b*), total color difference (ΔE*) calculated with respect to the white reference background (L* = 93.75, a* = 0.25, b* = −10.38), light transmission, Opa, moisture content (MC), water vapor permeability (WVP), OP, and puncture strength (PS) were determined as described previously [31,32]. Puncture deformation (PD, mm) was determined as the probe displacement at the moment of film puncture and used as a measure of film extensibility. The antioxidant activity was estimated using the 2,2′-azino-bis(3-ethylbenzothiazoline-6-sulfonic acid) radical cation (ABTS^*^^+^)-based assay [33] with slight modifications. Namely, film samples (2 × 2 cm) were immersed in 10 mL of ABTS^*+^ solution (absorbance ≈ 0.70 at 734 nm, 25 °C) under continuous stirring (500 rpm) for 45 min. The antiradical activity was expressed as the percentage of ABTS^*+^ scavenged, with the time required to reduce the ABTS^*+^ concentration by 50% (t_ABTS50%_).

A commercial PA/PE film (thickness ≈ 90 μm) was used as the reference material. Depending on the analysis, the number of replicates was three for WVP, OP, and antiradical activity, five for optical properties and MC, and eight for mechanical properties.

### 2.4. Preparation of Sliced CCPL, Coating Procedure, Packaging and Storage

Pork loins were divided into ~1 kg portions and cured with a curing mixture (99.5% sodium chloride, 0.5% sodium nitrite) added at 2.2% of the meat weight. The loins were stored at 4 °C for 24 h, cooked to an internal temperature of 70 °C, cooled in cold water to 20–25 °C, and sliced into 3-mm-thick slices. The slices, taken from refrigerated storage at 4 °C, were immersed in the FCFSs at ~40 °C for 10 min. The FCFS temperature was monitored and maintained at ≥30 °C to prevent premature GEL gelation and ensure uniform coating formation. The coated slices were then suspended and allowed to dry for 30 min under ambient conditions (approximately 22 °C and 50% RH). Uncoated slices served as controls and were not subjected to immersion or drying. Subsequently, all slices were vacuum-packaged in sterile PA/PE bags in groups of 15 (~200 g), with individual slices separated to prevent direct contact, and stored at 4 °C for 21 days. Analyses were performed on days 1, 7, 14, and 21 of storage.

### 2.5. Analysis of Sliced CCPL

#### 2.5.1. Initial (Before Packaging) Characteristics

FCFS uptake was calculated as the mass gain of CCPL slices after immersion and subsequent drying until the coating had solidified. The thickness of the methylene-blue-stained GEL coatings on CCPL was measured from microscopic images captured using a Leica M205C microscope equipped with a Leica DFC500 camera (Leica Microsystems, Wetzlar, Germany) and analyzed using AxioVision 4.8 software (Carl Zeiss, Jena, Germany) [34]. Cross-sectional samples were excised using a scalpel from three locations on each coated slice: the center and the upper and lower edges. Surface gloss of CCPL slices was measured at a 60° angle using a NHG268 glossmeter (3nh, Guangzhou, China). Surface pH was measured using a flat-surface electrode (Elmetron EPX-3, Zabrze, Poland) connected to a pH meter. Before measurement, 20 µL of deionized water was applied to the surface.

Sensory characteristics of CCPL samples were evaluated by a panel of 10 trained staff members. The selection and training of panel members followed ISO 8586:2023 [35]. Overall appearance and color acceptability were rated on a five-point scale (1 = lowest, 5 = highest acceptability), whereas gloss, odour, taste, and hardness were rated for intensity (1 = lowest, 5 = highest), in accordance with ISO 4121:2003 [36].

#### 2.5.2. Physicochemical and Microbiological Characteristics During Refrigerated Storage

Color parameters (L*, a*, and b*) were determined using an 8200 spectrophotometer (X-RITE Inc., Grand Rapids, MI, USA) with a 13-mm aperture, D65 illuminant, and 10° standard observer. ΔE* was calculated using Equation (1):

(1) $${∆E}^{*}=\;\sqrt{{∆L}^{*2}+{∆a}^{*2}+{∆b}^{*2}}$$

where Δ represents the difference between the color parameters of the uncoated and coated sample.

The water activity (a_w_) was measured at 20 °C using a LabMaster-aw water activity analyzer (NOVASINA AG, Lachen, Switzerland). The pH was determined at 20 °C using a CPC-501 pH meter (Elmetron, Zabrze, Poland) equipped with an ERH-111 electrode (Hydromet, Gliwice, Poland) after homogenizing 10 g of sample with 100 mL of distilled water. Thiobarbituric acid reactive substances (TBARS) content was determined spectrophotometrically according to the method described by Pikul et al. [37] and expressed as mg malondialdehyde (MDA) per kg of sample. Total viable counts (TVCs) were determined according to ISO 4833-1:2013-12 [38]. *Listeria monocytogenes* and *Salmonella* were analyzed according to ISO 11290-2:2017-07 [39] and ISO 6579-1:2017-04/A1:2020-09 [40], respectively. Samples for microbiological analyses were collected from the center of the cross-section of CCPL slices.

### 2.6. Statistical Analysis

Data on FCFS and film properties and on the coating experiment are presented as means ± standard deviations (*n* ≥ 3). Differences among the means for FCFS and film properties were assessed using one-way ANOVA. For the coating experiment, two-way ANOVA was performed with coating treatment and storage time as factors. Fisher’s test was used for post hoc comparisons. Sensory scores were analysed using the Friedman test for related samples, followed by pairwise Wilcoxon signed-rank tests with Holm adjustment for multiple comparisons. Sensory data are presented as median and interquartile range (Q1–Q3). Statistical significance was set at *p* < 0.05. Analyses were performed using Statistica 13.3 (TIBCO Software Inc., Palo Alto, CA, USA).

## 3. Results and Discussion

### 3.1. Comparison of the Properties of 5%-GEL and 10%-GEL FCFSs

**Figure 1:**
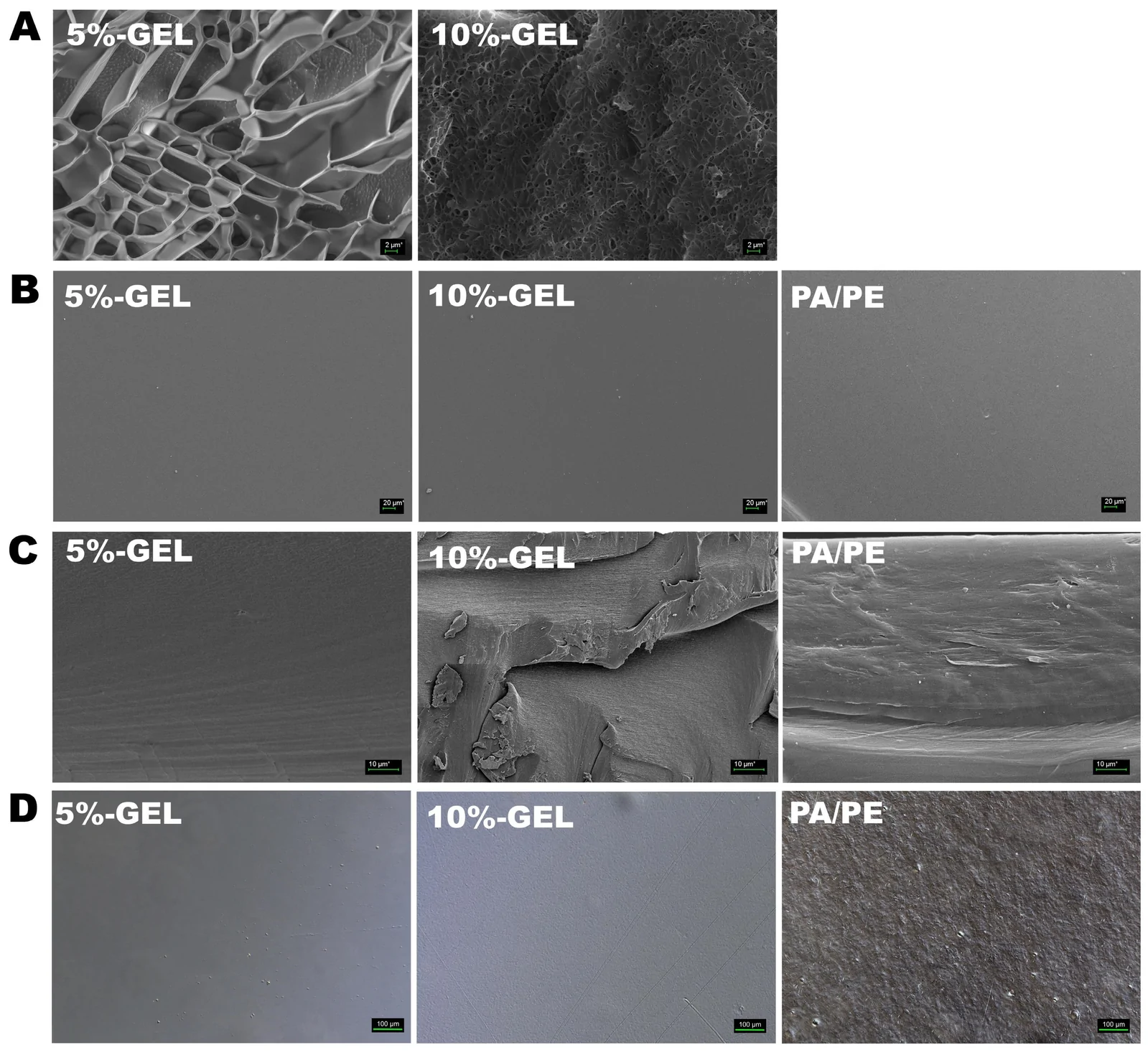
Cryogenic scanning electron microscopy (cryo-SEM) images of 5%- and 10%-gelatin (GEL) film/coating-forming solutions at 5000× magnification (**A**). Scanning electron microscopy (SEM) surface (500×, (**B**)), SEM cross-sectional morphology (2500×, (**C**)), and differential interference contrast microscopy images (100×, (**D**)) of 5%-GEL, 10%-GEL, and polyamide/polyethylene (PA/PE) films.

Cryo-SEM showed that increasing GEL concentration resulted in a more compact FCFS network with smaller pores (Figure 1A), corresponding to spaces left by frozen water. Van Vlierberghe et al. [41] observed a similar concentration-dependent reduction in pore size, which they attributed to an increased nucleation rate and restricted heat and mass transfer at higher GEL concentrations, limiting ice crystal growth.

**Figure 2:**
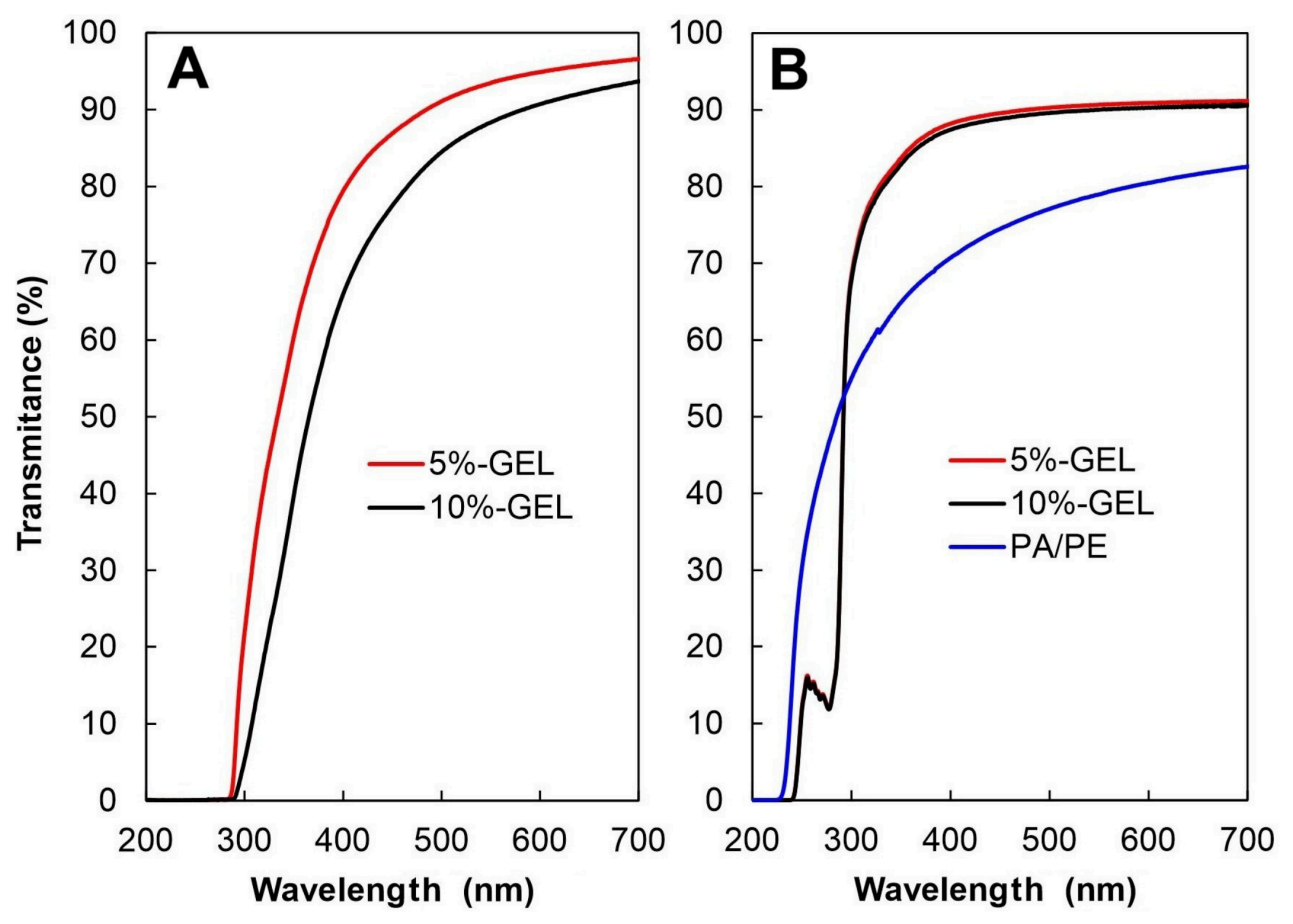
Light transmittance of 5%- and 10%-gelatin (GEL) film/coating-forming solutions (**A**) and of 5%-GEL, 10%-GEL, and polyamide/polyethylene (PA/PE) films (**B**).

Small but statistically significant differences (*p* < 0.05) were observed in the pH (5.03 vs. 5.09 at 40 °C) and lightness (27.06 vs. 26.16) of the 5%- and 10%-GEL FCFSs (Table 1). The observed pH values are consistent with those previously reported [22,42]. As expected, doubling the GEL concentration resulted in an FCFS with a higher chroma value, greater viscosity (22.97 vs. 9.10 mPa·s), a shorter gelation time (~25 vs. 55 min) (Table 1), and lower UV/VIS transmittance (Figure 2A). Both formulations equally and completely blocked UVC light (≤280 nm). Furthermore, doubling the GEL concentration resulted in more than a fourfold increase in GS (34.28 vs. 147.40 g, Table 1), reflecting stronger intermolecular interactions between GEL chains, consistent with the more compact network observed by cryo-SEM (Figure 1A). Similar increases in GEL gel strength with increasing GEL concentration have been reported previously [43].

### 3.2. Comparative Characterization of 5%-GEL, 10%-GEL, and PA/PE Films

Because the same amount of FCFS solids was applied per unit area, the resulting GEL films had similar thicknesses (~90 μm), matching that of the PA/PE film (Table 2). Nevertheless, it should be noted that GEL films are expected to swell with increasing RH, resulting in a significant increase in thickness, as reported previously [44].

**Table 2:** Thickness, color parameters (lightness, L*; redness, a*; yellowness, b*; and total color difference, ΔE*), opacity (Opa), and antioxidant activity (t_ABTS50%_, time required for 50% ABTS^*+^ reduction) of 5%-gelatin (GEL), 10%-GEL, and polyamide/polyethylene (PA/PE) films conditioned at 50% relative humidity.

Parameter	5%-GEL	10%-GEL	PA/PE
Thickness (μm)	87.20 ± 1.48 ^a^	91.25 ± 6.19 ^a^	89.20 ± 0.84 ^a^
L*	93.93 ± 0.19 ^b^	93.74 ± 0.16 ^b^	93.22 ± 0.07 ^a^
a*	0.21 ± 0.18 ^b^	0.05 ± 0.14 ^b^	−0.28 ± 0.07 ^a^
b*	−9.02 ± 0.11 ^b^	−8.96 ± 0.18 ^b^	−9.48 ± 0.08 ^a^
ΔE*	0.96 ± 0.13 ^b^	0.96 ± 0.15 ^b^	0.69 ± 0.15 ^a^
Opa (A_600_/mm)	0.48 ± 0.02 ^a^	0.48 ± 0.03 ^a^	1.06 ± 0.05 ^b^
t_ABTS50%_ (min)	8.3 ± 0.3 ^a^	7.8 ± 0.5 ^a^	n.e.

Different superscript letters within a row indicate significant differences (*p* < 0.05). n.e.—not estimable.

#### 3.2.1. Structural Properties

The GEL films exhibited no noticeable differences in microstructure, apart from minor variations in fracture surface roughness caused by the fracture process. Although both the GEL and PA/PE films exhibited smooth and homogeneous surfaces (Figure 1B), SEM revealed marked differences in their cross-sectional morphologies (Figure 1C). Consistent with previous reports, the GEL films exhibited dense and compact fracture surfaces [23,44], whereas the PA/PE film displayed the distinct layered structure typical of multilayer materials. DIC microscopy confirmed the homogeneous morphology of the GEL films and the heterogeneous morphology of the PA/PE film (Figure 1D), corroborating the SEM cross-sectional observations (Figure 1C) and previous findings [23,45,46]. The film microstructures remained essentially unchanged across the tested RH range.

**Figure 3:**
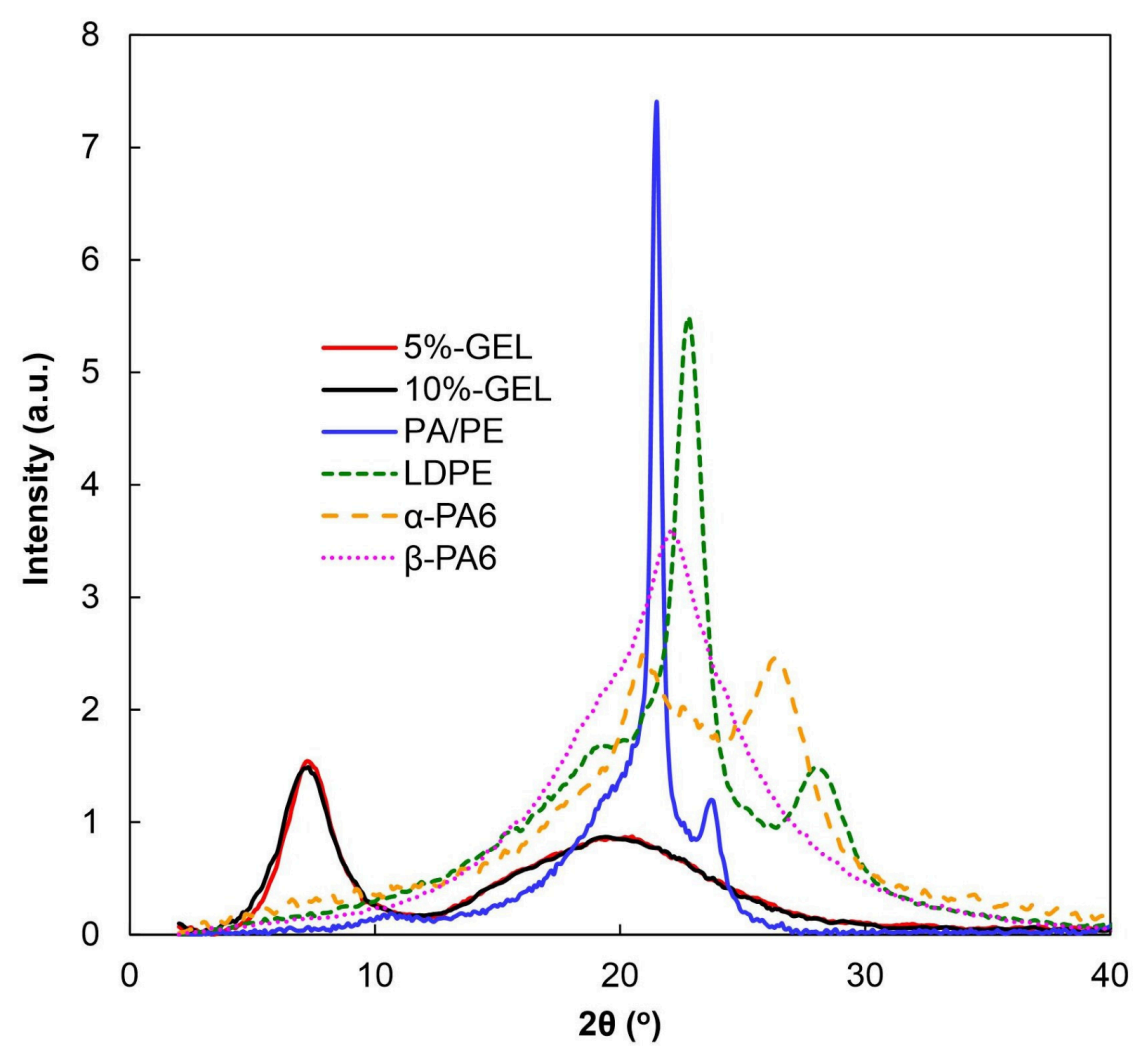
Wide-angle X-ray diffraction patterns of 5%-gelatin (GEL), 10%-GEL, and polyamide/polyethylene (PA/PE) along with the reference materials: low-density polyethylene (LDPE), α-PA6, and β-PA6 conditioned at 50% relative humidity.

The WAXD patterns of the 5%- and 10%-GEL films (Figure 3) were essentially similar, exhibiting a diffraction peak at 2θ ≈ 7.2°, attributed to triple-helix crystalline structures, together with a broad amorphous halo centered at 2θ ≈ 20.5° [32]. These results indicate that the approximately twofold higher initial hydration level of the GEL (18.8 vs. 8.8 g H_2_O/g for the 5%- and 10%-GEL FCFSs, respectively) and the associated differences in FCFS drying time had no significant effect on the final degree of renaturation, which was primarily governed by the identical GEL/glycerol ratio (5:1) and the processing conditions.

Reference materials, including low-density polyethylene (LDPE) and the α- and β-polymorphic forms of PA6, representing the PE and PA components of the multilayer PA/PE structure, respectively, were analyzed to identify the origin of the crystalline reflections in the PA/PE WAXD pattern. The PA/PE film exhibited a sharp and intense diffraction peak at 2θ ≈ 21.5° together with a weaker reflection at 23.7°, consistent with the diffractogram characteristic of the LDPE phase (Figure 3, [47]) constituting the outer layer of the material [48,49]. Notably, the intense peak at 2θ ≈ 21.5° partially overlaps with the diffraction range characteristic of PA6. Since WAXD probes the entire film thickness, the overlapping diffraction signals from the individual layers most likely reflect the relatively low PA content, which is typical of PA/PE films [49], and consequently the minor contribution of the PA layer to the overall crystallinity. In summary, the well-defined sharp diffraction signals suggest a higher degree of molecular ordering and crystallinity in the PA/PE film compared with the predominantly amorphous GEL films.

#### 3.2.2. Optical Properties

Although differences in optical properties were observed between FCFSs prepared with different GEL concentrations (Table 1), these differences were not reflected in the optical properties of the corresponding films (Table 2, Figure 2). This can be attributed to the fact that the films were prepared using the same amount of GEL/plasticizer solids per unit casting area, resulting in the same film thicknesses (Table 2) and, consequently, the same light absorption and scattering effects. This outcome is contrary to that of Nur Hanani et al. [29] who reported darker and more yellowish films at higher GEL concentrations. The differences observed by these authors may, however, have been related to the increase in film thickness with increasing GEL concentration.

Both GEL-based films exhibited higher lightness than the PA/PE film but also showed a more yellowish-red coloration, as indicated by their higher a* and b* values. Consequently, the GEL-based films would likely be more visually noticeable than the PA/PE film on light-colored product surfaces (ΔE = 0.96 vs. 0.69; Table 2).

Regarding color, no significant differences in light-barrier properties were found between the 5%- and 10%-GEL films (Figure 2B). The heterogeneous multilayer structure of the PA/PE film (Figure 1C,D) provided multiple light-scattering centers, resulting in lower transmittance in the visible (400–700 nm) region and, consequently, higher opacity (Opa; Table 2) and superior protection against UVA radiation (315–400 nm) (59–71% vs. 76–88%; Figure 2B) compared with the homogeneous GEL films (Figure 1C,D). The enhanced light attenuation may also have been influenced by the presence of impurities and structural defects within the PA component, as previously reported in the literature [50]. The UVA-blocking property is advantageous for food packaging applications, as UVA radiation accounts for ~95% of the UV radiation reaching the Earth’s surface [51] and can induce the formation of free radicals that promote the oxidation of food components [52]. Nevertheless, UVA has a lower photodestructive potential than UVB (which constitutes the remaining ~5% of terrestrial UV radiation) and UVC (100–280 nm) [51]. In this context, the GEL films exhibited more favourable UV-protective properties than the PA/PE film because of their superior blocking of short-wavelength UV radiation (Figure 2B). This behavior can be attributed to the presence of intrinsic UV-absorbing chromophores in GEL, such as tyrosine, phenylalanine, and disulfide bonds, with absorption maxima at approximately 275, 258, and 260 nm, respectively [53,54]. Consistent with the literature [23], an absorption peak at ~278 nm was observed in the GEL films (Figure S1), arising from overlapping contributions of the previously mentioned chromophores. For clarity, the absence of the peaks in the FCFS spectra (Figure 2A) can be attributed to stronger UV absorption resulting from the substantially longer optical path length (10 mm) compared with that of the films (~90 μm), leading to near-zero transmittance. Complete blocking of deep-UV radiation by the GEL films can be attributed to strong absorption by peptide bonds, with a major absorption band centered at approximately 190 nm and a weaker band in the 210–220 nm region [54]. The same effect in the PA/PE film was most likely attributable to the presence of amide groups in the PA component [50]. RH had no significant effect on the optical properties of the films.

#### 3.2.3. MC and WVP

**Figure 4:**
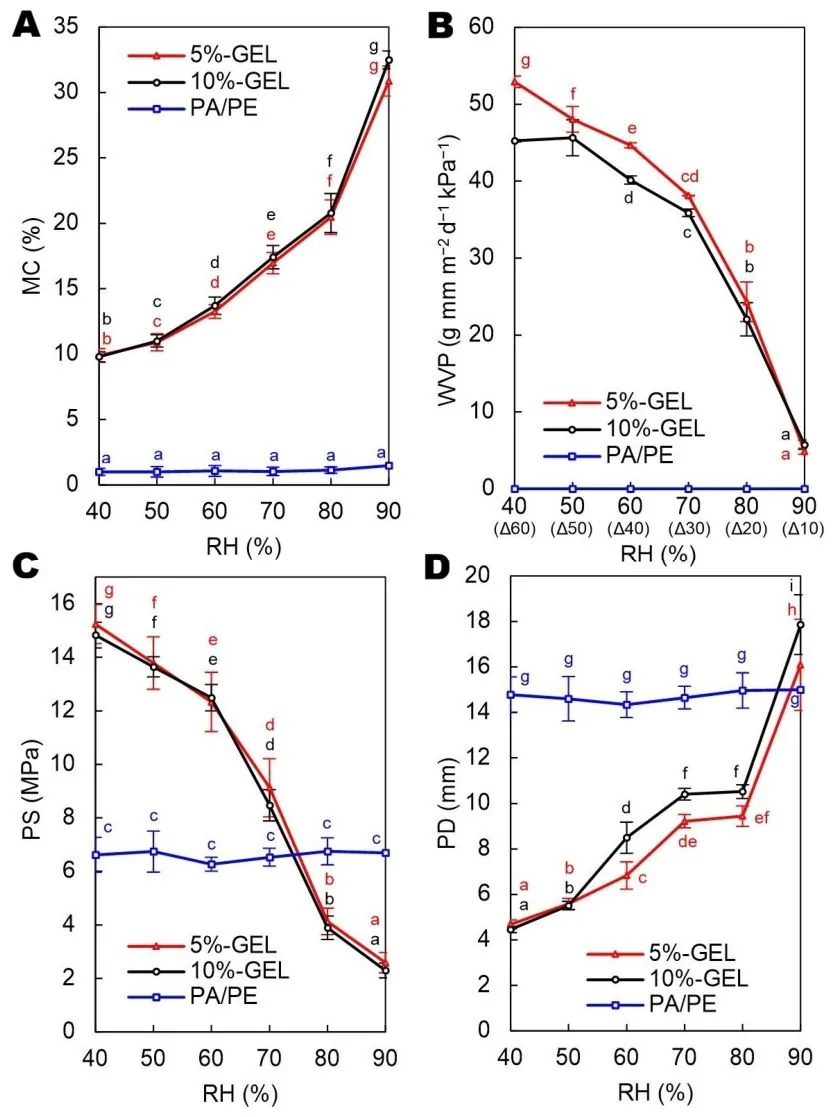
Effect of relative humidity (RH) on moisture content (MC) (**A**), water vapor permeability (WVP) (**B**), puncture strength (PS) (**C**), and puncture deformation (PD) (**D**) of 5%-gelatin (GEL), 10%-GEL, and polyamide/polyethylene (PA/PE) films. Values from Δ10 to Δ60 denote the RH gradient across the film. Different superscript letters indicate significant differences (*p* < 0.05).

The GEL-based films exhibited similar MC values, increasing steadily with RH from ~10% at 40% RH to ~32% at 90% RH (Figure 4A), consistent with a previous report [44]. A particularly sharp increase occurred between 80% and 90% RH, reflecting the high swelling capacity of GEL under high-humidity conditions. Because the hydrophobic outer PE layers [55] effectively shielded the hydrophilic PA layer [56] from moisture, the MC of the PA/PE film remained low (1–1.5%) throughout the entire RH range.

Given that 100% RH was maintained inside the cups, the WVP of the GEL-based films exhibited a clear decreasing trend with increasing external RH as a result of the reduction in the water vapor pressure gradient across the film (Figure 4B). Specifically, increasing the RH from 40% to 90% reduced the WVP from 52.91 to 4.88 g mm m^−2^ d^−1^ kPa^−1^ (≈11-fold) for the 5%-GEL film and from 45.25 to 5.73 g mm m^−2^ d^−1^ kPa^−1^ (≈8-fold) for the 10%-GEL film. The WVP values measured under the Δ50 condition were within the range previously reported for GEL-based films [24,29,46,57]. Interestingly, at RH ≤ 60% (i.e., at higher RH gradients: Δ40–Δ60), the 10%-GEL film exhibited lower WVP values than the 5%-GEL film. As no significant differences in the structural properties of the GEL films were detected by SEM and WAXD (Figure 1B–D and Figure 2), the slightly improved water vapor barrier performance (5.0–14.5%) under high driving forces may be related to subtle differences in film microstructure formed during drying. Previous studies have shown that drying conditions can influence the organization of GEL networks, including the formation of intermolecular bonds and the preservation of helical structures, which may affect the barrier, swelling, and mechanical properties of GEL films [58,59]. Therefore, although not directly confirmed by the applied techniques, it can be hypothesized that the 10%-GEL film (obtained by shorter drying) may have retained a denser and more highly entangled polymer matrix (Figure 1A) due to the closer proximity of GEL molecules in the more concentrated FCFS. Such a structure could increase resistance to water vapor transport by creating more tortuous diffusion pathways compared with the initially more relaxed, open network of the 5%-GEL matrix (Figure 1A). This interpretation is consistent with the concept discussed by Jopling [59], who proposed that closer packing of GEL molecules at higher solution concentrations may favor intermolecular bond formation during drying. However, at RH ≥ 70%, extensive water sorption likely disrupted polymer–polymer interactions, increased chain mobility, and expanded free volume [60], leading to pronounced water-induced plasticization that diminished the influence of GEL concentration on WVP (Figure 4B). Nevertheless, the molecular mechanisms underlying these effects remain unclear and warrant further investigation.

As expected, the PA/PE film, owing to the hydrophobic nature of its polyolefin component [61], exhibited no detectable WVP regardless of the RH gradient (Figure 4B).

#### 3.2.4. OP

Under moderate moisture conditions (40–60% RH), the GEL-based films exhibited statistically similar OP values, which were approximately 4- to 30-fold lower than those of the PA/PE film, depending on the RH level (Table 3). This finding is consistent with the well-established fact that biopolymer-based films, including GEL-based films, generally exhibit superior oxygen barrier properties compared with conventional petroleum-based packaging polymers, although this advantage is mainly maintained under low to intermediate RH conditions [62]. The superior oxygen barrier properties of dry GEL films stem from their compact, polar matrix, which limits oxygen diffusion due to the low affinity between the hydrophilic GEL network and non-polar oxygen molecules [63], in contrast to the apolar and highly gas-permeable PE matrix [64]. As shown in Figure 1C, unlike the more heterogeneous layered structure of the PA/PE film, the GEL films displayed a compact and homogeneous cross-sectional morphology, thereby hindering oxygen diffusion.

Although a clear increasing trend in OP values with increasing RH was observed for the GEL films (Table 3), the differences were not statistically significant due to the considerable variability within the dataset. When the RH exceeded 60%, absorbed water molecules plasticized the GEL films by disrupting interchain hydrogen bonds, increasing polymer chain mobility and free volume, and consequently enhancing oxygen diffusion through the film, leading to a pronounced deterioration in oxygen barrier performance [19]. At 70% and 80% RH, the films exhibited unstable behaviour, resulting in sinusoidal instrument readings. Measurements could not be completed at 90% RH because of moisture-induced deformation of the films. In contrast, due to its hydrophobic nature and limited moisture uptake (Figure 4A), the PA/PE film maintained measurable OP values across the entire RH range, with OP generally showing a gradual increase as RH increased.

It is worth noting that the OP values of the GEL films were lower than those reported previously [23]. This difference may be attributed to the lower plasticizer content (20% vs. 40% based on GEL), which caused less disruption of protein–protein interactions and promoted the formation of a denser protein network, consequently limiting oxygen diffusion [65,66].

**Table 3:** Oxygen permeability (cm^3^ µm m^−2^ d^−1^) of 5%-gelatin (GEL), 10%-GEL, and polyamide/polyethylene (PA/PE) films measured at different relative humidity (RH) levels.

RH (%)	5%-GEL	10%-GEL	PA/PE
40	157.97 ± 10.76 ^a^	127.75 ± 62.93 ^a^	4234.06 ± 573.64 ^b^
50	347.06 ± 20.64 ^a^	372.95 ± 37.15 ^a^	4513.78 ± 709.83 ^b^
60	1195.09 ± 46.32 ^a^	1231.25 ± 224.01 ^a^	5056.72 ± 939.82 ^bc^
70	2853.33–6350.00 ^~^	3010.67–7726.67 ^~^	6126.23 ± 1317.19 ^c^
80	9908.33–19,454.17 ^~^	10,778.33–22,281.67 ^~^	7986.08 ± 2049.15 ^d^
90	n.e.	n.e.	15,333.02 ± 1699.42 ^e^

Different superscript letters within a row indicate significant differences (*p* < 0.05). ~—unstable instrument readings. n.e.—not estimable.

#### 3.2.5. Mechanical Properties

Although the 5%-GEL and 10%-GEL FCFSs differed significantly in terms of GS (Table 1), the PS of the resulting films remained comparable across the entire range of RH (Figure 4C). Consistent with the literature [62], increasing RH reduced the mechanical strength of the GEL films because absorbed water (Figure 4A) acted as a plasticizer, weakening intermolecular hydrogen bonding and reducing film matrix cohesion. Up to 70% RH, the GEL films exhibited PS values up to 2.3-fold higher than those of the PA/PE film (9.12–15.24 MPa vs. ~6.6 MPa; Figure 4C). At higher RH, however, extensive moisture uptake (Figure 4A) markedly reduced film cohesion, lowering the PS to approximately 2.5 MPa at 90% RH. Nevertheless, this value remained comparable to those reported for glycerol-plasticized pea protein isolate, gum Arabic, corn starch, and sorbitol-plasticized soy protein isolate films conditioned at standard 50% RH (PS = ~3.00, 2.61, 2.14, and 2.20 MPa respectively) [22,25,57,67] and exceeded that of chitosan lactate films (PS = 1.45 MPa) [68]. Because all the aforementioned values were obtained using the same puncture test methodology, these comparisons indicate the inherently strong and cohesive protein network formed by GEL, even in its wet state.

The PD of the GEL-based films increased markedly with increasing RH, from approximately 4.6 mm at 40% RH to 16–18 mm at 90% RH (Figure 4D), indicating a pronounced plasticizing effect of water. Although both films exhibited the same PS (Figure 4C), they reached failure through different deformation behavior. At low-to-medium RH, both films exhibited similar rigidity due to strong interchain hydrogen bonding. At RH ≥ 60%, however, the 10%-GEL films consistently exhibited higher PD values than the 5%-GEL films, reflecting a more ductile mechanical response. Because the GEL films did not differ in microstructure (Figure 1B–D), crystalline order (Figure 3), or MC (Figure 4A), their different deformability may reflect subtle variations in chain entanglement density and free volume that remain undetectable by standard characterization methods but become significantly amplified upon water-induced plasticization at elevated RH levels. The longer drying time may have allowed the 5%-GEL films to form a more ordered and rigid molecular network that restricted chain slippage, resulting in lower PD. Conversely, the 10%-GEL films, which underwent shorter drying, presumably featured a less organized network structure that allowed greater macromolecular displacement and higher deformability upon plasticization. The hydrophobic nature of the PA/PE film and its negligible moisture uptake (Figure 4A) resulted in no RH-dependent changes in the mechanical properties (Figure 4C,D).

#### 3.2.6. Antioxidant Properties

The GEL films exhibited similar ABTS^*+^ scavenging activity (Figure S2, Table 2), which was not affected by RH. Consistent with previous findings, the observed antioxidant capacity is attributed to the electron-donating ability of amino acid residues in GEL [46,69,70]. Interestingly, the PA/PE film also exhibited radical-scavenging activity, although its contribution was negligible relative to the GEL films (~2.5% vs. ~92%; Figure S2). This minor activity may result from chemical stabilizers, including antioxidants, incorporated during film manufacture [71,72].

### 3.3. Comparison of the Effects of 5%-GEL and 10%-GEL Coatings on the Quality of CCPL

#### 3.3.1. Effect of Coatings on Initial (Before Packaging) Characteristics of CCPL

**Figure 5:**
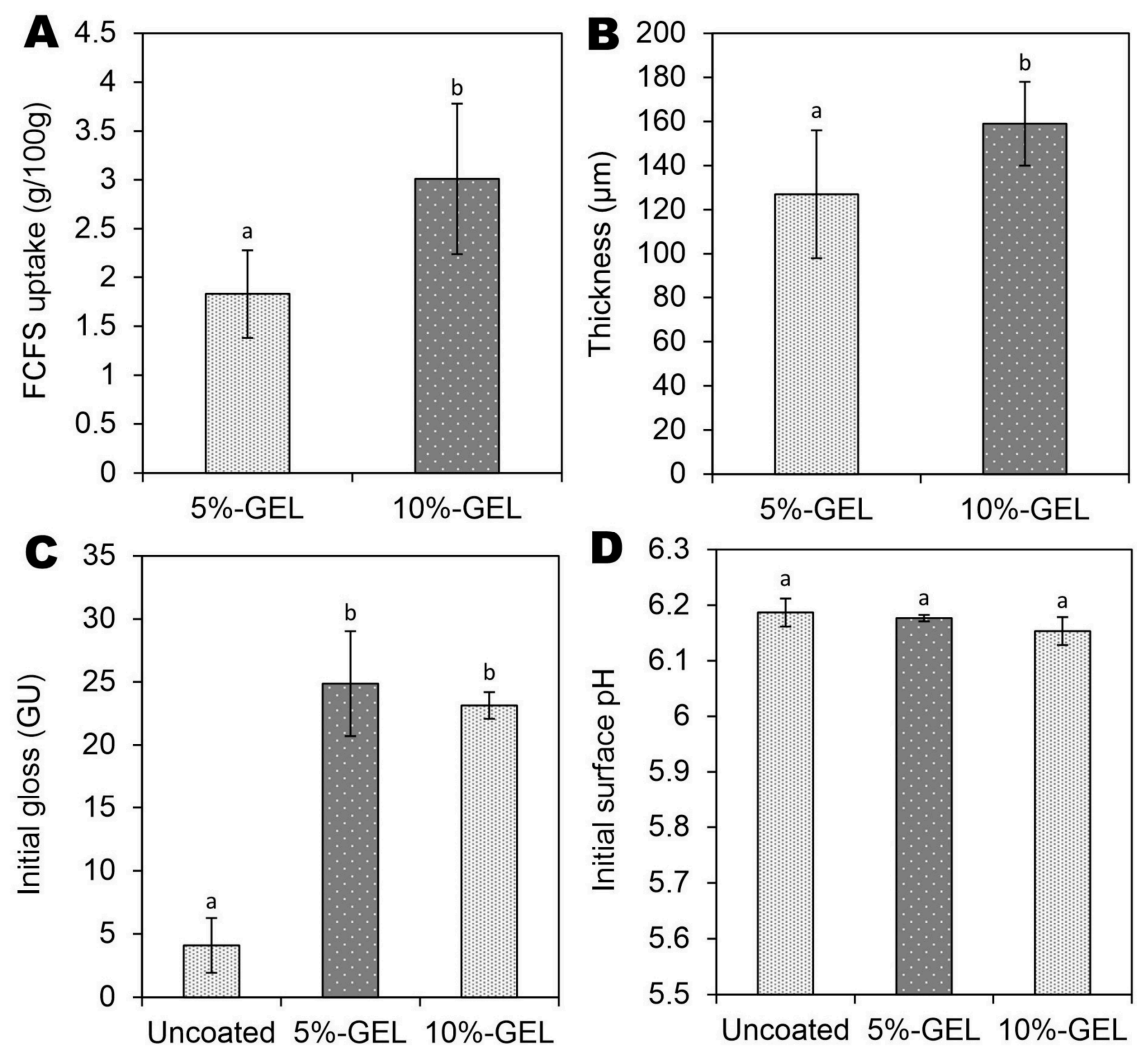
Effect of gelatin (GEL) concentration on film/coating-forming solution (FCFS) uptake (**A**), initial (before packaging) coating thickness (**B**), gloss (**C**), and surface pH (**D**) of cooked cured pork loin. Different superscript letters indicate significant differences (*p* < 0.05).

**Figure 6:**
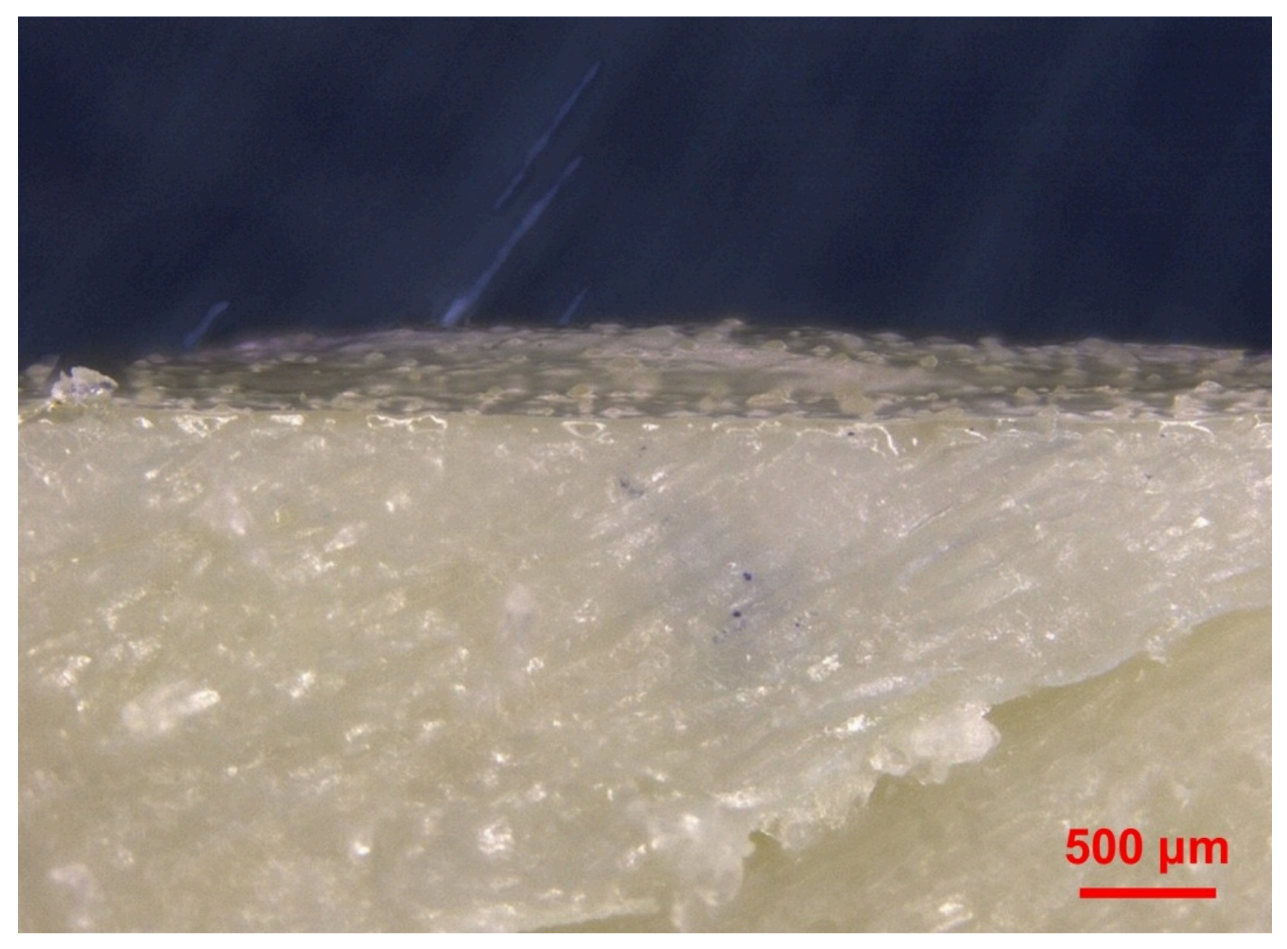
Representative micrograph of the 10%-GEL coating on cooked cured pork loin at 25× magnification.

The higher viscosity of the 10%-GEL FCFS (Table 1) resulted in greater FCFS uptake during immersion (3.01 vs. 1.83 g/100 g; Figure 5A) and a thicker coating (159 vs. 128 µm; Figure 5B). The coatings remained adherent to the CCPL, with no visible peeling during sample slicing, although their thickness appeared uneven (Figure 6).

Surface gloss was significantly higher in both GEL-coated treatments than in uncoated CCPL, with no significant difference between 5%-GEL and 10%-GEL (Figure 5C). Surface pH did not differ significantly between treatments (Figure 5D).

Compared with the control, both GEL-coated treatments received significantly lower scores for overall appearance (median 3 vs. 4.5) and color acceptability (median 3 vs. 4) (Table S1). The coated CCPL also exhibited higher perceived gloss intensity (median 5 vs. 3, consistent with the instrumental gloss measurements (Figure 5C)), lower odour intensity (median 3 vs. 4), lower taste intensity (median 4 vs. 5), and higher perceived hardness (median 4 vs. 2). No significant differences were found between the 5% and 10% GEL treatments for any of the attributes. Lower acceptability of the GEL-coated CCPL slices may reflect their uncharacteristically glossy appearance (Figure S3). In turn, the lower perceived odour and taste intensities may be due to the aroma-barrier properties of GEL [73] and/or the transfer of flavour compounds from the product surface into the FCFS during immersion. As shown in Figure S4, substantial leaching of components from CCPL into the FCFS was evident. The higher perceived hardness of GEL-coated CCPL may be associated with the high Bloom strength (240 Bloom) of the GEL forming the coating. The higher GS of the 10%-GEL FCFS (Table 1) did not translate into greater perceived hardness of CCPL (Table S1).

#### 3.3.2. Effect of Coatings on Color

The two-way ANOVA showed that coating treatment significantly affected L* and a*, whereas b* was not significantly affected by treatment (Table S2). Storage time significantly affected a* and b*, but not L*. No significant treatment × storage time interaction was observed for any of the individual color parameters (Table S2). In contrast, ΔE* was significantly affected by coating treatment and, importantly, by the treatment × storage time interaction, whereas the main effect of storage time was not significant (Table S2). This interaction demonstrates that the evolution of total color difference over time followed distinct patterns depending on the coating concentration (5%-GEL vs. 10%-GEL).

While the uncoated and 5%-GEL-coated samples darkened over time, samples coated with 10%-GEL maintained stable L* values, resulting in significantly higher lightness at the end of storage (Table 4). Generally, the 10%-GEL-coated CCPL exhibited lower redness (a*) than the uncoated samples, likely reflecting the greater visual masking effect of the coating formed from the more concentrated GEL solution rather than changes in meat pigment stability. Despite minor fluctuations during storage, b* values remained comparable among treatments, suggesting that the coatings had little effect on yellowness. Based on the established ΔE* thresholds [74], the color differences between the 5%-GEL-coated and uncoated CCPL samples were generally below the perceptibility threshold for an untrained observer (1 < ΔE* < 2). The only exception was one sample with a ΔE* of 2.73 (Table 4), for which the color difference was considered noticeable (2 < ΔE* < 3.5). In contrast, the 10%-GEL coating resulted in clearly distinguishable (3.5 < ΔE* < 5) color differences at some storage times (Table 4).

#### 3.3.3. Effect of Coatings on a_w_, pH, TBARS Content, and TVC

The two-way ANOVA showed significant effects of coating treatment and storage time on a_w_, but no significant interaction between these factors (Table S2). In contrast, coating treatment, storage time, and their interaction significantly affected pH, TBARS, and TVC (Table S2), indicating that their changes during storage differed among treatments.

a_w_ remained high throughout storage, ranging from 0.968 to 0.984 (Table 5), which is typical of cooked meat products due to their high MC [75,76]. During the first week of storage, both GEL coatings resulted in similarly higher a_w_ values than the uncoated control (*p* < 0.05), suggesting that the coating process introduced additional free water, most likely at the product surface. After 14 and 21 days of storage, no significant differences were observed among treatments, indicating that the free water associated with the coatings had likely redistributed within the product and reached equilibrium.

The pH values of CCPL ranged from 6.08 to 6.35 (Table 5), consistent with previously reported values [75,77]. The pH increase during storage in uncoated CCPL may have been associated with microbial activity and the formation of basic compounds during protein degradation, including biogenic amines, as reported for cooked cured pork products [78]. Although GEL-coated CCPL showed lower pH values than the control (6.08–6.29 vs. 6.17–6.35), this difference cannot be directly attributed to the acidity of FCFS (pH ≈ 5; Table 1), as the surface pH of CCPL did not differ significantly between treatments before packaging (Figure 5). Moreover, the absence of a pH increase in coated samples despite their higher microbial counts (Table 5) suggests that pH changes were not directly related to TVC. The observed differences may simply reflect natural pH variability rather than a direct effect of the coatings.

TBARS content ranged from 0.015 to 0.180 mg MDA/kg (Table 5). On day 1, both GEL-coated CCPL samples exhibited lower TBARS values than the uncoated control, with the lowest value observed for the 5%-GEL treatment (0.023 mg MDA/kg). The uncoated control maintained consistently low TBARS values throughout storage (0.015–0.080 mg MDA/kg). In contrast, TBARS levels in the GEL-coated samples increased markedly during storage, reaching 0.119 and 0.180 mg MDA/kg at the end of storage in the 5%- and 10%-GEL treatments, respectively. These results indicate that the GEL coatings not only failed to retard lipid oxidation during storage but also reduced the oxidative stability of the product. This unexpected result, particularly considering the antioxidant properties of the GEL films (Table 2, Figure S2), contrasts with previous reports showing that GEL-based coatings generally reduce [18,79] or at least do not affect TBARS [14,80] in fresh or thermally processed meat. Nevertheless, an increase in TBARS values is not without precedent, as has also been reported for refrigerated fish fillets coated with calcium-cross-linked sodium alginate [81]. One possible explanation is that the coating procedure itself increased the exposure of the CCPL to oxygen before vacuum packaging, thereby facilitating oxidative changes. Alternatively, interference of GEL degradation products with the TBARS assay cannot be excluded, potentially leading to an overestimation of lipid oxidation [82]. Thus, the observed TBARS pattern (Table 5) may reflect genuine oxidative changes associated with increased oxygen exposure during coating application, interference of GEL degradation products with the TBARS assay, or a combination of both mechanisms. However, the present data do not allow these possibilities to be distinguished. Therefore, the observed increase in TBARS should be interpreted with caution.

**Table 5:** Effect of 5%- and 10%-gelatin (GEL) coatings on water activity (a_w_), pH, thiobarbituric acid reactive substance (TBARS) content, and total viable count (TVC) of sliced cooked cured pork loin vacuum-packaged in polyamide/polyethylene bags during refrigerated storage.

Parameter	Coating	Storage Time (Days)			
		1	7	14	21
a_w_	Uncoated	0.974 ± 0.008 ^a^	0.975 ± 0.004 ^a^	0.968 ± 0.001 ^a^	0.972 ± 0.001 ^a^
	5%-GEL	0.984 ± 0.004 ^b^	0.980 ± 0.001 ^b^	0.976 ± 0.001 ^a^	0.976 ± 0.002 ^a^
	10%-GEL	0.983 ± 0.007 ^b^	0.979 ± 0.002 ^b^	0.976 ± 0.001 ^a^	0.976 ± 0.001 ^a^
pH	Uncoated	6.17 ± 0.099 ^a^	6.35 ± 0.008 ^c^	6.31 ± 0.022 ^c^	6.22 ± 0.039 ^b^
	5%-GEL	6.24 ± 0.010 ^a^	6.23 ± 0.016 ^a^	6.11 ± 0.005 ^a^	6.08 ± 0.010 ^a^
	10%-GEL	6.12 ± 0.013 ^a^	6.29 ± 0.015 ^b^	6.18 ± 0.026 ^b^	6.12 ± 0.006 ^a^
TBARS (mg MDA/kg)	Uncoated	0.080 ± 0.013 ^c^	0.015 ± 0.002 ^a^	0.022 ± 0.002 ^a^	0.042 ± 0.003 ^b^
	5%-GEL	0.023 ± 0.003 ^a^	0.155 ± 0.032 ^e^	0.160 ± 0.007 ^e^	0.119 ± 0.001 ^d^
	10%-GEL	0.051 ± 0.003 ^b^	0.162 ± 0.047 ^e^	0.120 ± 0.005 ^d^	0.180 ± 0.009 ^f^
TVC (log CFU/g)	Uncoated	4.40 ± 0.02 ^a^	6.15 ± 0.04 ^c^	7.04 ± 0.03 ^d^	7.32 ± 0.04 ^e^
	5%-GEL	4.41 ± 0.03 ^a^	7.08 ± 0.05 ^d^	8.00 ± 0.04 ^f^	7.92 ± 0.05 ^f^
	10%-GEL	5.43 ± 0.03 ^b^	6.92 ± 0.06 ^d^	7.95 ± 0.05 ^f^	7.80 ± 0.04 ^f^

Different superscript letters within a row indicate significant differences (*p* < 0.05). MDA- malondialdehyde, CFU—colony forming units (log-transformed before statistical analysis).

During refrigerated storage, progressive microbial growth was observed in all treatments; however, GEL-coated CCPL samples reached higher TVCs than the control (Table 5). It should be noted that the uncoated control did not undergo immersion and drying before vacuum packaging. Thus, the differences may reflect several factors, including microbial introduction with the FCFSs, handling outside refrigerated conditions, and the presence of GEL as an additional nutrient source [83]. Moreover, the additional water introduced with the FCFSs and retained within the PA/PE packaging may have locally increased surface moisture, thereby creating more favorable conditions for microbial proliferation. However, since all samples exhibited high a_w_ values (>0.96), the slightly higher initial a_w_ of the GEL-coated samples (Table 5) was unlikely to be a major factor contributing to the observed differences in TVC. Except on day 1, no significant differences in TVCs were found between the 5%-GEL and 10%-GEL coatings.

During refrigerated storage, *Listeria monocytogenes* counts in all CCPL samples remained below the enumeration limit of the applied method (<10 CFU/g), whereas *Salmonella* was not detected in 25 g of product. The non-detection of these pathogens is reassuring but does not, by itself, confirm the microbiological safety of CCPL.

### 3.4. Limitations of the Study

Several limitations should be considered when interpreting the results of this study. First, performing the complete set of analytical determinations at the exact time of vacuum packaging (day 0) was not physically feasible within the experimental workflow. Therefore, the first measurements were performed on day 1, which served as a post-packaging baseline. However, this time point cannot fully replace measurements taken on day 0, immediately after packaging. Consequently, differences observed between treatments in a_w_, TBARS, and TVC may have already arisen during coating application and drying, or resulted from initial chemical and microbiological changes occurring during the 1-day storage period. Second, the uncoated control was not subjected to immersion and drying, whereas the GEL-coated samples underwent both procedures. Therefore, the initial differences in evaluated parameters cannot be attributed exclusively to the presence of GEL, as they may also reflect the effects of the coating procedure itself. Future studies should include an appropriate immersion/drying control and measurements immediately before or after vacuum packaging, where technically feasible, to distinguish the effects of GEL from those associated with coating application.

## 4. Conclusions

Despite clear differences in the properties of the 5%- and 10%-GEL FCFSs, the resulting films, prepared using the same amount of GEL/plasticizer solids per unit casting area and therefore having comparable thicknesses, exhibited no significant structural differences and shared most physicochemical properties. Compared with the 5%-GEL film, however, the 10%-GEL film showed improved water vapor barrier properties under ΔRH 40–60% conditions and lower deformability at RH ≥60%. These differences may be related to subtle molecular-scale variations in the polymer network that were not resolved by the applied characterization techniques but became apparent under specific humidity conditions.

Regardless of GEL concentration, the GEL coatings reduced the acceptability of CCPL, possibly due to increased gloss, leaching of flavor and aroma compounds, and greater perceived hardness. Although GEL films provided substantially better oxygen barrier properties than PA/PE at 40–60% RH, this advantage progressively diminished with increasing humidity and was no longer evident at 70% RH. Despite their inherent antiradical activity, GEL films did not show a protective effect on the oxidative stability of CCPL, as assessed by TBARS. The higher TBARS values observed in GEL-coated samples during storage may have resulted from increased oxygen exposure during coating application before vacuum packaging and/or interference of GEL degradation products with the TBARS assay. The present data do not allow these possibilities to be distinguished. The higher microbial counts in GEL-coated CCPL may have resulted from the greater availability of nutrients and free water associated with the hydrophilic GEL coating and/or microbial contamination during application.

Overall, the findings demonstrate that the favorable oxygen-barrier and antioxidant properties of GEL films under standardized testing conditions did not translate into preservation benefits in high-moisture meat products. The faster deterioration of vacuum-packaged sliced CCPL suggests that the practical performance of GEL coatings depends not only on their intrinsic film properties but also on coating application and food–coating interactions.

Future studies should investigate the incorporation of antioxidants and antimicrobial agents, together with optimized coating procedures that minimize oxygen exposure and microbial contamination.

## Figures and Tables

**Table 1:** Effect of gelatin (GEL) concentration on the pH, color parameters (lightness, L*; chroma, C; and hue angle, h), opacity (Opa), dynamic viscosity (η), gelation time (t_gel_), gelation temperature (T_gel_), and gel strength (GS) of film/coating-forming solutions.

Parameter	5%-GEL	10%-GEL
pH at 25 °C	5.36 ± 0.02 ^a^	5.40 ± 0.03 ^b^
pH at 40 °C	5.03 ± 0.02 ^a^	5.09 ± 0.01 ^b^
L*	27.06 ± 0.02 ^b^	26.16 ± 0.07 ^a^
C	1.61 ± 0.11 ^a^	2.53 ± 0.08 ^b^
h (°)	58.97 ± 2.55 ^b^	51.02 ± 1.71 ^a^
Opa (A_600_/mm)	0.0023 ± 0.0003 ^a^	0.0042 ± 0.0005 ^b^
η (mPa·s)	9.10 ± 0.20 ^a^	22.97 ± 0.38 ^b^
t_gel_ (min)	55.00 ± 1.00 ^b^	24.57 ± 0.58 ^a^
T_gel_ (°C)	25.37 ± 0.35 ^a^	27.43 ± 0.40 ^b^
GS (g)	34.28 ± 3.00 ^a^	147.40 ± 9.54 ^b^

Different superscript letters within a row indicate significant differences (*p* < 0.05).

**Table 4:** Effect of 5%- and 10%-gelatin (GEL) coatings on the lightness (L*), redness (a*), yellowness (b*), and total color difference (ΔE*) of sliced cooked cured pork loin vacuum-packaged in polyamide/polyethylene bags during refrigerated storage.

Parameter	Coating	Storage Time (Days)			
		1	7	14	21
L*	Uncoated	74.92 ± 2.49 ^b^	76.1 ± 2.26 ^b^	75.59 ± 2.79 ^b^	73.3 ± 3.01 ^a^
	5%-GEL	76.31 ± 1.6 ^b^	75.22 ± 2.86 ^b^	73.27 ± 1.28 ^a^	72.31 ± 3.17 ^a^
	10%-GEL	76.45 ± 1.14 ^b^	78.16 ± 1.34 ^b^	75.30 ± 1.07 ^b^	77.13 ± 2.12 ^b^
a*	Uncoated	6.79 ± 0.88 ^a^	6.90 ± 0.49 ^a^	7.06 ± 0.76 ^a^	7.57 ± 1.07 ^a^
	5%-GEL	5.49 ± 0.81 ^b^	6.01 ± 1.16 ^a^	6.50 ± 1.18 ^a^	7.40 ± 1.63 ^ab^
	10%-GEL	5.41 ± 0.83 ^b^	4.17 ± 1.10 ^b^	6.13 ± 0.83 ^a^	6.31 ± 0.70 ^b^
b*	Uncoated	10.26 ± 0.47 ^a^	9.74 ± 0.43 ^b^	9.34 ± 0.34 ^b^	9.05 ± 0.55 ^ab^
	5%-GEL	10.58 ± 1.00 ^a^	9.87 ± 0.71 ^b^	10.66 ± 1.11 ^a^	10.00 ± 1.22 ^a^
	10%-GEL	10.71 ± 0.55 ^a^	11.13 ± 1.16 ^a^	10.75 ± 0.66 ^a^	8.64 ± 0.73 ^b^
ΔE*	5%-GEL	1.93 ± 0.48 ^b^	1.26 ± 0.31 ^a^	2.73 ± 0.61 ^bc^	1.38 ± 0.45 ^a^
	10%-GEL	2.12 ± 0.74 ^b^	3.69 ± 0.74 ^c^	1.57 ± 0.43 ^ab^	4.06 ± 0.76 ^c^

Different superscript letters within a row indicate significant differences (*p* < 0.05). ΔE* represents the difference between the color parameters of the uncoated and coated samples.

## Data Availability

The original contributions presented in this study are included in the article and its Supplementary Materials. Further inquiries can be directed to the corresponding author.

## Supplementary Materials

The following supporting information can be downloaded at https://www.mdpi.com/article/10.3390/polym18212790/s1, Figure S1: Absorbance spectra of 5%-gelatin (GEL), 10%-GEL, and polyamide/polyethylene (PA/PE) films; Figure S2: Kinetics of ABTS^*+^ scavenging by 5%-gelatin (GEL), 10%-GEL, and polyamide/polyethylene (PA/PE) films; Figure S3. Effect of gelatin (GEL) coatings on the appearance of cooked cured pork loin slices before vacuum packaging; Figure S4. Appearance of the film/coating-forming solutions before and after 10 min of contact with the cooked cured pork loin; Table S1. Effect of gelatin (GEL) coatings on the initial sensory characteristics of cooked cured pork loin slices before vacuum packaging; Table S2: *p*-values for the effects of coating treatment, storage time, and their interaction on the quality parameters of sliced cooked cured pork loin vacuum-packaged in polyamide/polyethylene bags during refrigerated storage.

## Abbreviations

The following abbreviations are used in this manuscript:

η	Dynamic viscosity
ΔE*	Total color difference
ABTS	2,2′-azino-bis(3-ethylbenzothiazoline-6-sulfonic acid)
ABTS^*+^	2,2′-azino-bis(3-ethylbenzothiazoline-6-sulfonic acid) radical cation
ANOVA	Analysis of variance
a_w_	Water activity
CCPL	Cooked cured pork loin
FCFS	Film/coating-solution
DIC	Differential interference contrast
CFU	Colony-forming unit
GS	Gel strength
GEL	Gelatin
LDPE	Low-density polyethylene
MC	Moisture content
Opa	Opacity
OP	Oxygen permeability
PA/PE	Polyamide/polyethylene
PS	Puncture strength
PD	Puncture deformation
RH	Relative humidity
SEM	Scanning electron microscopy
t_ABTS50%_	Time required for 50% ABTS^*+^
TBARSs	Thiobarbituric acid reactive substances
T_gel_	Gelation temperature
t_gel_	Gelation time
TVC	Total viable count
WAXD	Wide-angle X-ray diffraction
WVP	Water vapor permeability
