# Supplementary material for: Effect of Gelatin Concentration on Humidity-Dependent Film Properties and Coating Performance: A Case Study of Vacuum-Packaged Sliced Cooked Cured Pork Loin
Source: Polymers (Basel). 2026 Nov 8;18(21):2790. doi: 10.3390/polym18212790 (PMC13765967; doi:10.3390/polym18212790)
Supplement: Supplementary file 1 [file polymers-18-02790-s001.zip › Suplementary file.pdf]

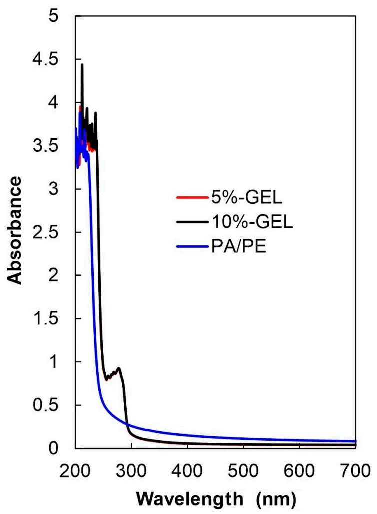

Figure S1. Absorbance spectra of 5%-gelatin (GEL), 10%-GEL, and polyamide/polyethylene (PA/PE) films.

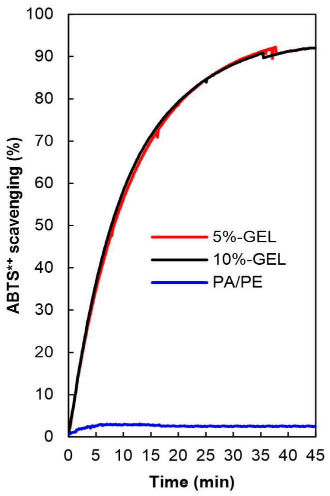

Figure S2. Kinetics of ABTS•+ scavenging by 5%-gelatin (GEL), 10%-GEL, and polyamide/polyethylene (PA/PE) films.

Table S1. p-values for the effects of coating treatment, storage time, and their interaction on the quality parameters of sliced cooked cured pork loin vacuum-packaged in polyamide/polyethylene bags during refrigerated storage.

Parameter	Coating treatment	Storage time	Coating treatment × Storage time
L*	0.032	0.148	0.502
a*	0.003	0.025	0.617
b*	0.067	0.012	0.156
ΔE*	<0.001	0.202	<0.001
a_w	<0.001	0.002	0.758
pH	<0.001	<0.001	<0.001
TBARS	<0.001	<0.001	<0.001
TVC	<0.001	<0.001	<0.001

p-values were obtained using two-way ANOVA. Differences were considered statistically significant at $p < 0.05$.

Table S2. Effect of gelatin (GEL) coatings on the initial sensory characteristics of cooked cured pork loin slices before vacuum packaging.

Sensory attribute	Uncoated	5%-GEL	10%-GEL	p-value
Overall appearance	4.5 (4–5)[a]	3 (3–4)[b]	3 (3–4)[b]	0.00053
Color acceptability	4 (4–5)[a]	3 (3–3.75)[b]	3 (3–3)[b]	0.000075
Gloss intensity	3 (3–4)[a]	5 (5–5)[b]	5 (5–5)[b]	0.00012
Odour intensity	4 (4–4)[a]	3 (3–3)[b]	3 (3–3)[b]	0.00016
Taste intensity	5 (4.25–5)[a]	4 (3.25–4)[b]	4 (3–4)[b]	0.00011
Hardness intensity	2 (2–3)[a]	4 (4–4)[b]	4 (4–4)[b]	0.000045

Values are presented as medians (Q1–Q3). The last column shows the p-values of the Friedman test ($p < 0.05$ indicates statistically significant differences among treatments). Different superscript letters within a row indicate significant differences ($p < 0.05$), based on pairwise Wilcoxon signed-rank tests with Holm adjustment.

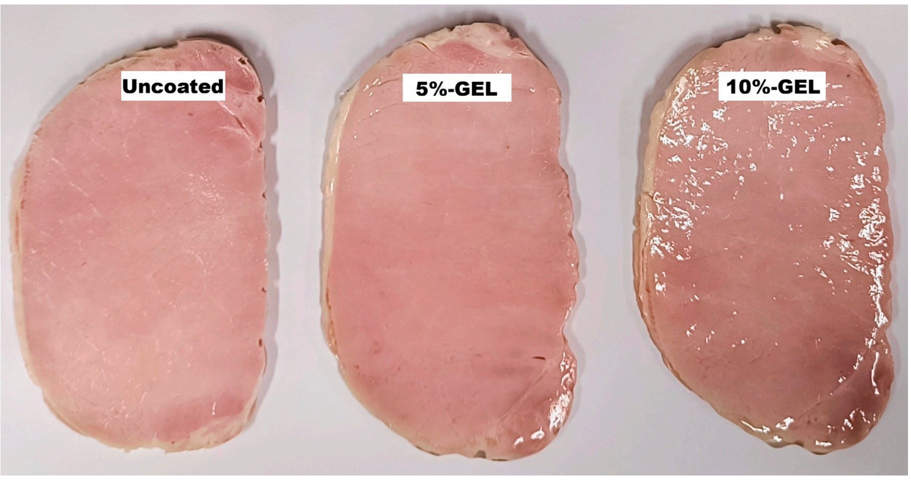

Figure S3. Effect of gelatin (GEL) coatings on the appearance of cooked cured pork loin slices before vacuum packaging.

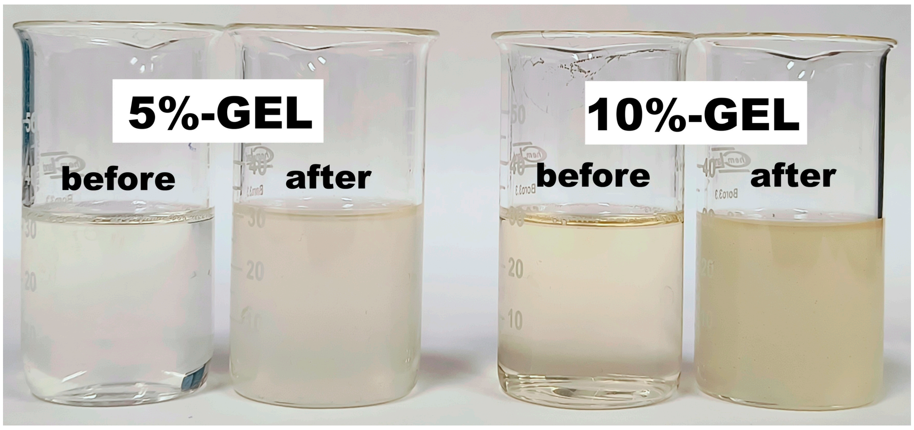

Figure S4. Appearance of the film/coating-forming solutions before and after 10 min of contact with the cooked cured pork loin.
